# Vision and hearing impairments, cognitive impairment and mortality among long-term care recipients: a population-based cohort study

**DOI:** 10.1186/s12877-016-0286-2

**Published:** 2016-05-27

**Authors:** Kazuko Mitoku, Naoko Masaki, Yukiko Ogata, Kazushi Okamoto

**Affiliations:** Department of Community Nursing, Graduate School of Nursing, University of Human Environments, 3-220, Ebata-cho, Obu City, Aichi 444-0035 Japan; Department of Community Nursing, Graduate School of Nursing, Japanese Red Cross Hiroshima College of Nursing, 1-2, Ajinadai-higashi, Hatsukaichi City, Hiroshima 738-0052 Japan; Department of Community Nursing, Faculty of Nursing, Fukuoka Prefectural University, 4395, Ita-cho, Tagawa City, Fukuoka 825-8585 Japan; Department of Epidemiology, Graduate School of Nursing and Health, Aichi Prefectural University, Togoku, Kamishidami, Moriyama-ku, Nagoya, Aichi 463-8502 Japan

**Keywords:** Elders, Visual impairment, Hearing impairment, Cognitive impairment, Death

## Abstract

**Background:**

Vision and hearing impairments among elders are common, and cognitive impairment is a concern. This study assessed the association of vision and hearing impairments with cognitive impairment and mortality among long-term care recipients.

**Methods:**

Data of 1754 adults aged 65 or older were included in analysis from the Gujo City Long-Term Care Insurance Database in Japan for a mean follow-up period of 4.7 years. Trained and certified investigators assessed sensory impairments and cognitive impairment using a national assessment tool. Five-level scales were used to measure vision and hearing impairments. Cognitive performance was assessed on two dimensions, namely communication/cognition and problem behaviors. We performed logistic regression analysis to estimate odd ratios (ORs) and 95 % confidence intervals (CIs) for the association of vision and hearing impairments with cognitive impairment. Using Cox proportional hazard regression models, we obtained hazard ratios (HRs) for mortality.

**Results:**

Of 1754 elders, 773 (44.0 %) had normal sensory function, 252 (14.4 %) vision impairment, 409 (23.3 %) hearing impairment, and 320 (18.2 %) dual sensory impairment. After adjusting for potential cofounders, ORs of cognitive impairment were 1.46 (95 % CI 1.07–1.98) in individuals with vision impairment, 1.47 (95 % CI 1.13–1.92) in those with hearing impairment, and 1.97 (95 % CI 1.46–2.65) in those with dual sensory impairment compared to individuals with normal sensory function. The adjusted HR of overall mortality was 1.29 (95 % CI 1.01–1.65) in individuals with dual sensory impairment and cognitive impairment relative to normal sensory and cognitive functions.

**Conclusions:**

Cognitive impairment was most common in individuals with dual sensory impairment, and those with dual sensory impairment and cognitive impairment had increased mortality.

## Background

Most elders experience age-related changes, including vision and hearing impairments. Sensory impairments affect elders’ everyday lives, including independence [1, 2], social participation [3, 4], health-related quality of life [5, 6], and mental health [7]. The prevalence of cognitive impairment also increases among elders and is a concern [8]. As people become more disabled and increasingly dependent on others with age, assisting elders may be challenging for communities.

Previous studies have reported a possible association between vision impairment and cognitive function in older populations [9–12]. Vision impairment was associated with cognitive impairment in women aged 69 or older who had osteoporotic fractures [9]. Regarding specific eye diseases, age-related macular degeneration, a leading cause of irreversible vision loss, was associated with cognitive impairment [10]. Furthermore, elders with visual impairment, particularly those with visual impairment due to cataract, were more likely to have cognitive impairment in Asian populations aged 60–80 [11], while only a weak correlation was observed in Australian populations aged 50 and older [12].

Hearing impairment and cognitive decline among elders have a clear link [12–16]. Hearing impairment accelerates cognitive impairment [14] and is an independent risk factor for dementia [13]. However, impairment in hearing function is gradual and often under-recognized [13, 15] and consequently has received minimal attention [6]. Dual sensory impairment is prevalent between 9 % and 21 % of elders over age 70, and the prevalence increases with age [17]. Dual sensory impairment is associated with cognitive impairment [9, 18], and cognitive function declines more quickly, particularly in socially disengaged elders [19].

Japan is experiencing unprecedented aging among the population. By 2035, the proportion of elders is projected to comprise 33.4 % of the total population in Japan such that one in three people will be aged 65 or older [20]. In 2000, Japan introduced a universal social long-term care insurance system for people aged 65 or over and some people aged 40–64 with specific disabilities to receive home and community-based services besides universal medical insurance [21]. The insured or family must apply to the municipal government for needs assessment. Trained and certified investigators conduct on-site survey using a 79-item need-assessment tool on physical and mental status. The results are analyzed by computer to generate a preliminary assessment. The certification committee, consisting of five health and welfare professionals, reviews the survey results and the primary physician’s opinion for a final eligibility decision.

The relationship between sensory impairments and cognitive impairment has been reported [9–19], but the relationship among sensory impairments, cognitive impairment and mortality remains unclear. Using the long-term care insurance data, we assessed the association of vision and hearing impairments with cognitive impairment and mortality among elders.

## Methods

### Design

This population-based cohort study used administrative health care data with a mean follow-up period of 4.7 years.

### Data source and study cohort

This study cohort was drawn from the Gujo City Long-Term Care Insurance Data, which included all residents who were certified as new beneficiaries in Gujo City, Gifu Prefecture, Japan, between April 1, 2003 and December 31, 2004. The level of dependency is determined as a result of eligibility assessment; low level (home-bound, requiring help to go out), medium level (bed-bound, requiring some assistance but able to maintain a sitting position), or high level (totally bed-bound, requiring full-time care).

Subjects of the study were community-dwelling elders aged 65 years or older in Gujo City; required assistance or care in community settings; and were eligible for long-term care insurance. Of 2338, 584 elders who were classified with a high level of dependency (totally bed-bound) were excluded and 1754 who were classified as having a low or middle level of dependency were included in the analysis. The participants were followed until March 31, 2009.

### Data collection

The Gujo City Long-Term Care Insurance Data include the results of preliminary and secondary eligibility assessments.

### Definition

We operationally defined “cognitive impairment” as “any cognitive impairment from mild to severe including dementia assessed by the trained and certified investigator using the functional assessment for cognitively impaired elders” in this study.

### Measurements

We used the data on vision and hearing impairments and cognitive impairment collected from the eligibility assessment process for long-term care insurance. Trained and certified investigators assessed vision and hearing impairments and cognitive impairment using the national assessment tool for determining eligibility for long-term care insurance [21, 22].

Vision at baseline was determined according to five levels: “normal sight,” “able to see a visual acuity chart at a distance of 1 m,” “able to see a visual acuity chart in front,” “very little sight,” and “indeterminable due to communication difficulty.” For analysis, “able to see a visual acuity chart at a distance of 1 m” and “able to see a visual acuity chart in front” were merged to form one value—“able to see an object near the front of the eyes.” Hearing at baseline was determined according to five levels: “normal hearing,” “barely hear normal conversation,” “barely hear loud conversation,” “barely hear,” and “indeterminable due to communication difficulty.” For analysis, “barely hear normal conversation” and “barely hear loud conversation” were merged to form one value—“able to hear loud conversation.”

Cognitive performance was assessed on two dimensions, namely communication/cognition and problem behaviors, using the functional assessment measures for cognitively impaired elders. The assessment measures include communication, short-term memory, location awareness and understanding of daily tasks, remembering own name and date of birth, and recognizing the season of year in addition to 18 problem behaviors (e.g., wandering, agitation and resistance to care). The levels of cognitive impairment were none, mild (requiring assistance due to symptoms/behavior related to cognitive impairment or communication difficulty), moderate (requiring care due to symptoms/behavior or communication difficulties), and severe (requiring specialized care due to significant psychiatric symptoms). Cognitive impairment in this study was determined by its presence or absence according to this classification.

Demographic information including age, sex and level of dependency was obtained during the preliminary eligibility assessment. Moving away from the study area (certification of residence) and death (death certificate) during follow-up were ascertained by Local Civil Registry. The 10th Revision of the International Classification of Diseases (ICD-10) code was used to define the diagnoses.

### Analysis

We performed logistic regression to calculate unadjusted and adjusted odd ratios (ORs) and 95 % confidence intervals (CIs) to assess the association of baseline vision and hearing impairments with cognitive impairment. The analysis was cross-sectional. Cox proportional hazard regression models were used to estimate unadjusted and adjusted hazard ratios (HRs) for mortality during the follow-up period of 4.7 years. Potential covariates, including age, sex, level of dependency and comorbidities, were included in the models. Comorbidities included diabetes, neurological disorders (all diseases of the nervous system in the ICD-10 Chapter IV G00-G99 except Alzheimer’s disease, e.g., Parkinson’s disease, cerebellar degeneration and amyotrophic lateral sclerosis), hypertension, heart disease, cerebrovascular disease, respiratory disease, musculoskeletal and connective tissue disorders, and eye and ear diseases. We classified the participants into three age groups (65–74, 75–89, 90 or older) in consideration of average life expectancy among Japanese (80.21 years old for men; 86.61 for women) [23]. Chi-square test, Mann-Whitney *U* test and *t*-test were used to compare baseline characteristics. Fisher’s exact test was used for small sample sizes. *P* values less than 0.05 were considered significant.

## Results

### Study population

Baseline characteristics of the 1754 study participants (605 men and 1149 women) are presented in Table 1. The mean age of men and women was 80.89 (standard deviation [SD] 7.39) years and 82.42 (SD 6.95) years, respectively. Women were older than men (*p* < 0.001). The participants with a low level of dependency were more likely to be women (67.4 % vs. 56.4 %; *p* < 0.001). With respect to underlying diseases for long-term insurance eligibility, men had more neurological disorders except Alzheimer’s disease (*p* = 0.043), heart disease (*p* = 0.009), cerebrovascular disease (*p* < 0.001), and respiratory disease than women (*p* < 0.001), while women had more hypertension (*p* = 0.043) and musculoskeletal and connective tissue disorders than men (*p* < 0.001). There was no significant difference in baseline cognitive impairment between men and women (52.7 % vs. 54.9 %).

**Table 1:** Participant characteristics

			Men		Women		Total		*p*	
			*n*	%	*n*	%	*n*	%		
	Total		605	100.0	1149	100.0	1754	100.0		
Mean age (SD) (years)			80.89 (7.39)		82.42 (6.95)				< .001	^f^
Age group (years)		65–74	127	21.0	160	13.9	287	16.4	< .001	^e^
		75–89	400	66.1	789	68.7	1189	67.8		
		≥90	78	12.9	200	17.4	278	15.8		
Level of dependency^a^	Low		341	56.4	774	67.4	1115	63.6	< .001	^c^
	Medium		264	43.6	375	32.6	639	36.4		
Functional assessment^b^	Cognitive impairment		319	52.7	631	54.9	950	54.2	.382	^c^
Comorbidity (ICD10)	Diabetes		50	8.3	96	8.4	146	8.3	.513	
	Neurological disorders (except Alzheimer’s disease)		57	9.4	80	7.0	137	7.8	.043	^c^
	Hypertension		104	17.2	286	24.9	390	22.2	.294	
	Heart disease		50	8.3	60	5.2	390	6.3	.009	
	Cerebrovascular disease		200	33.1	204	17.8	404	23.0	< .001	
	Respiratory disease		64	10.6	61	5.3	125	7.1	< .001	
	Musculoskeletal connective tissue disorder		153	25.3	524	45.6	677	38.6	< .001	
	Eye disease		11	1.8	27	2.3	38	2.2	.294	^d^
	Ear disease		7	1.2	14	1.2	21	1.2	.556	
	Dementia		100	16.5	274	23.8	374	21.3	< .001	^c^

^a^Level of dependency (long-term care insurance)

^b^Functional assessment for cognitively impaired elders (long-term care insurance)

^c^ *χ* ^2^ test

^d^Fisher’s exact test

^e^Mann-Whitney *U* test

^f^ *t* test

Of the 1754 participants, 773 (44.1 %) had normal vision and hearing functions, 252 (14.4 %) had vision impairment, 409 (23.3 %) had hearing impairment, and 320 (18.2 %) had dual sensory impairments. There were no significant differences owing to sex among these four groups. Individuals with hearing impairment were 1.6-fold more than those with vision impairment (*p* < 0.001). About 70 % of participants had either vision or hearing impairment or both (dual sensory impairment) (Table 2).

**Table 2:** Prevalence of vision/hearing impairment

		Men		Women		Total	
		*n*	%	*n*	%	*n*	%
	Total	605	100.0	1149	100.0	1754	100.0
Vision/hearing functions	Normal vision/hearing function	252	41.7	521	45.3	773	44.1
	Vision impairment	79	13.1	173	15.1	252	14.4
	Hearing impairment	159	26.3	250	21.8	409	23.3
	Dual sensory impairment	115	19.0	205	17.8	320	18.2

### Association between sensory impairments and cognitive impairment (Table 3)

Of the 1754 participants, 950 (54.2 %) had cognitive impairment at baseline (Table 3). Cognitive impairment was found in 360 (46.5 %) individuals with normal vision and hearing function, 138 (54.8 %) with vision impairment, 237 (57.9 %) with hearing impairment, and 215 (67.2 %) with dual sensory impairment (*p* = 0.01). By age group, cognitive impairment increased with age: 107 individuals (37.3 %) aged 65–74, 668 (56.2 %) aged 75–89, and 175 (62.9 %) aged 90 or older (*p* = 0.021).

**Table 3:** Vision/hearing impairment and cognitive decline by age group

		Total	Cognitive decline	OR	Univariate analysis		*p*	OR	Multivariate analysis		*p*	
					95 % CI				95 % CI			
					Lower limit	Upper limit			Lower limit	Upper limit		
Overall	Normal vision/hearing function	773	360	1.000	-	-	-	1.000	-	-	-	^a^
	Vision impairment	252	138	1.389	1.04	1.85	0.02	1.457	1.07		0.02	
	Hearing impairment	409	237	1.581	1.24	2.01	<0.001	1.473			0.00	
	Dual sensory impairment	320	215	2.349	1.79	3.09	<0.001	1.965			<0.001	
	Total	1754	950									
65–74 (yrs)	Normal vision/hearing function	185	61	1.000	-	-	-	1.000	-	-	-	^b^
	Vision impairment	59	27	1.715	0.94	3.12	0.08	1.774	0.89	3.54	0.10	
	Hearing impairment	27	10	1.196	0.52	2.77	0.68	1.246	0.49	3.19	0.65	
	Dual sensory impairment	16	9	2.614	0.93	7.35	0.07	2.653	0.78	8.99	0.12	
	Total	287	107									
75–85	Normal vision/hearing function	518	258	1.000	-	-	-	1.000	-	-	-	^b^
	Vision impairment	172	99	1.367	0.97	1.94	0.08	1.513	1.04	2.21	0.03	
	Hearing impairment	281	164	1.413	1.05	1.89	0.02	1.584	1.15	2.18	0.00	
	Dual sensory impairment	218	147	2.086	1.50	2.91	<0.001	1.916	1.34	2.74	<0.001	
	Total	1189	668									
≥90	Normal vision/hearing function	70	41	1.000	-	-	-	1.000	-	-	-	^b^
	Vision impairment	21	12	0.943	0.35	2.53	0.91	0.976	0.32	2.99	0.97	
	Hearing impairment	101	63	1.173	0.63	2.19	0.62	1.222	0.60	2.49	0.58	
	Dual sensory impairment	86	59	1.546	0.80	2.99	0.19	1.437	0.68	3.04	0.34	
	Total	278	175									

^a^Adjusted for age, sex, level of dependency, diabetes, neurological disease, hypertension, heart disease, cerebrovascular disease, respiratory disease, musculoskeletal and connective tissue disorders, and eye and ear diseases

^b^Adjusted for the above variables except age

In univariate analysis, ORs of cognitive impairment were 1.389 (95 % CI 1.04–1.85) in those with vision impairment, 1.581 (95 % CI 1.24–2.01) in those with hearing impairment, and 2.349 (95 % CI 1.79–3.09) in those with dual sensory impairment. After adjustment for potential cofounders, the prevalence of cognitive impairment was higher in individuals with dual sensory impairment (1.965, 95 % CI 1.46–2.65), followed by those with hearing impairment (1.473, 95 % CI 1.13–1.92) and vision impairment (1.457, 95 % CI 1.07–1.98).

The prevalence of cognitive impairment was similar between individuals with vision impairment and those with hearing impairment. In univariate analysis by age group, the prevalence of cognitive impairment in the 75–89 age group was significantly higher among individuals with dual sensory impairment (2.086, 95 % CI 1.98–2.91), followed by those with hearing impairment (1.413, 95 % CI 1.055–1.90), and vision impairment (1.367, 95 % CI 0.97–1.94). Multivariate analysis showed that the prevalence of cognitive impairment in the 75–89 age group was significantly higher among individuals with dual sensory impairment (1.916, 95 % CI 1.339–2.742), followed by those with hearing impairment (1.584, 95 % CI 1.15–2.18) and vision impairment (1.513, 95 % CI 1.04–2.21).

### Association of mortality risk with sensory impairments and cognitive impairment (Table 4)

Univariate analysis showed that mortality risk was significantly high in individuals with cognitive impairment and normal vision and hearing functions (HR 1.496; 95 % CI 1.21–1.85). Vision impairment was not significantly associated with mortality regardless of the presence of cognitive impairment. Mortality risk in individuals with hearing impairment was significantly high for those with cognitive impairment (HR 1.889; 95 % CI 1.50–2.38) and without (HR 1.614; 95 % CI 1.24–2.09). Among individuals with dual sensory impairment, mortality risk was high in those with cognitive impairment (HR 1.980; 95 % CI 1.57–2.50) and without cognitive impairment (HR 1.644; 95 % CI 1.22–2.22). Multivariate analysis showed that HR of mortality was 1.290 (95 % CI 1.01–1.65) in individuals with dual sensory impairment and cognitive impairment, and 1.225 (95 % CI 0.96–1.56) in those with hearing impairment and cognitive impairment relative to normal sensory and cognitive functions.

**Table 4:** Vision/hearing impairment, cognitive decline and mortality by age group

		Cognitive decline	Participants				Univariate analysis				Multivariate analysis			
			95 % CI			HR	95 % CI		*p*	HR	95 % CI		*p*	
			Total	Death	Movers		Lower limit	Upper limit			Lower limit	Upper limit		
Overall	Normal vision/hearing function	No	413	155	11	1.00	-	-	-	1.000	-	-	-	^a^
		Yes	360	184	9	1.496	1.21	1.85	0.00	1.138	0.91	1.42	0.25	
	Vision impairment	No	114	46	2	1.060	0.76	1.47	0.73	1.043	0.74	1.46	0.81	
		Yes	138	60	2	1.096	0.81	1.48	0.55	0.875	0.65	1.19	0.39	
	Hearing impairment	No	172	90	4	1.614	1.24	2.09	0.00	1.154	0.88	1.51	0.30	
		Yes	237	138	9	1.889	1.50	2.38	0.00	1.225	0.96	1.56	0.10	
	Dual sensory impairment	No	105	58	3	1.644	1.22	2.22	0.00	1.106	0.81	1.51	0.52	
		Yes	215	133	8	1.980	1.57	2.50	0.00	1.290	1.01	1.65	0.04	
	Total		1754	864	48									

^a^Adjusted for age, sex, level of dependency, diabetes, neurological disease, hypertension, heart disease, cerebrovascular disease, respiratory disease, musculoskeletal and connective tissue disorders, and eye and ear diseases

## Discussion

This study provides a picture of sensory impairment, cognitive impairment and mortality among the long-term care recipients with mean age over 80 in the community. In the present cohort, about 70 % of elders had either vision or hearing impairment or dual sensory impairment, and hearing impairment outnumbered vision impairment. Both vision and hearing impairments were associated with cognitive impairment. Dual sensory impairment was the greatest risk factor for cognitive impairment, and those with dual sensory impairment and cognitive impairment had increased risk of mortality.

High prevalence of hearing impairment among elders [17, 24] and dual sensory impairment as a risk factor for cognitive impairment [9, 17, 18] in the literature were consistent with our results. In addition, individuals with sensory impairment may be more likely to have limited social participations and interactions with others and are thus more likely to have cognitive impairment [14, 18].

The elders with dual sensory impairment and cognitive impairment had increased mortality in our study. Sensory impairment increases not only risks of accidents and injuries but also risks of communication difficulty, social isolation and physical and psychological functional decline; these factors could negatively affect life expectancy [25]. Cognitive impairment is also a robust predictor for mortality [26], and even mild cognitive impairment is associated with increased mortality [27]. Increased risk of falls and poor control of underlying diseases due to cognitive impairment could reduce survival [27].

Gopinath et al. [25] reported that hearing impairment was an independent risk factor for mortality, and Fisher et al. [28] reported that not vision but hearing impairment increased risk of all-cause or cardiovascular disease-related mortality in men. In our study, however, the result of association between hearing impairment and mortality was not significant.

Hearing impairment is a leading type of disability worldwide [24]. Michikawa et al. [6] reported that elders with hearing impairment were more significantly associated with adverse health outcomes than those with vision impairment among the Japanese population. Given the high prevalence and burden of hearing impairment among elders, early detection, before vision or cognitive impairments occur, is important. Healthcare providers should also offer information about treatment and rehabilitation of sensory impairment to elders [1]. Because poor communication function may cause elders and healthcare providers to misunderstand their individual conditions and negatively affect outcomes, healthcare providers need to help elders maintain communication function. Previous studies suggest that cognitive impairment could be attenuated by the use of hearing aids [14] and cognitive rehabilitation through communication [15]. Some elders may not use hearing aids because of difficulty in tuning them; therefore, healthcare providers should instruct elders about appropriate use.

### Strengths and limitations

The strengths of the study included a population-based sample of community-dwelling elders, and few data were missing in the sample. Very few people changed their address, and therefore panel attrition was low (2.7 %). This study supports the evidence regarding the relationship of dual sensory impairments with cognitive impairment and mortality using the national assessment tool administered by trained and certified investigators. Given the high prevalence of multimorbid conditions in elders, comorbidities were adjusted in addition to age, sex and level of dependency.

The study had several limitations. The study population comprised long-term care recipients in a rural area of Japan; thus, the generalizability of the study is limited. The higher prevalence of cerebrovascular disease and cognitive impairment was a possible bias for higher cognitive impairment. In this population, more men had serious diseases such as heart disease, cerebrovascular disease and respiratory disease, while more women had musculoskeletal and connective tissue disorders. These differences could be related to the result that women had lower level of dependency. We used only baseline measurements of sensory impairments and cognitive impairment, and we did not examine subsequent functional changes in vision, hearing and cognition. Because the measures for cognitive function are unique to the assessment for long-term care insurance, “cognitive impairment” in the present study includes both mild cognitive impairment and dementia. In addition, no objective test was used to measure sensory impairments. Standardized objective sensory measurement tools are needed for elders [1].

## Conclusions

In the present study, about 70 % of the long-term care recipients had either vision or hearing impairment or dual sensory impairment, and hearing impairment was 1.6-fold more common than vision impairment. This study supports the relationship between dual sensory impairment and cognitive impairment, and increased risk of mortality in those with dual sensory impairment and cognitive impairment. With advancing age, people get frailer and become more disabled. The qualitative study, however, reveals that elders adopt better with their conditions and still can achieve quality of life despite late-life disability and dependence on others in daily lives [29]. Healthcare providers should be aware of the increased risk of cognitive impairment and mortality in those with sensory impairment. Further studies are needed whether early identification and management of sensory impairment could improve cognitive outcomes and survival in elders.

## Abbreviations

CI, confidence interval; HR, hazard ratio; ICD, International Classification of Diseases; OR, odds ratio

## References

[1] Haanes CG, Kirkevold M, Hofoss D, Eilertsen G (2015). Discrepancy between self-assessments and standardized tests of vision and hearing abilities in older people living at home: an ROC curve analysis. J Clin Nurs.

[2] Hajek A, Brettschneider C, Lange C, Plsselt T, Wiese B, Steinmann S (2015). Longitudinal predictors of institutionalization in old age. PloS ONE.

[3] Polku H, Mikkola TM, Rantakokko M, Portegijs E, Törmäkangas T, Rantanen T, Viljanen A (2015). Self-reported hearing difficulties and changes in life-space mobility among community-dwelling older adults: a two-year follow-up study. BMC Geriatr.

[4] Viljanen A, Törmäkangas T, Vestergaard S, Andersen-Ranberg K (2014). Dual sensory loss and social participation in older Europeans. Eur J Ageing.

[5] Eisele M, Kaduszkiewicz H, König H-H, Lange C, Wiese B, Prokein J (2015). Determinants of health-related quality of life in older primary care patients: results of the longitudinal observational AgeCoDe Study. Br J Gen Pract.

[6] Michikawa T, Nishiwaki Y, Kikuchi Y, Nakano M, Iwasawa S, Asakura K (2009). Gender-specific associations of vision and hearing impairments with adverse health outcomes in older Japanese: a population-based cohort study. BMC Geriatr.

[7] Zhang X, Bullard KM, Cotch MF, Wilson MR, Rovner BW, McGwin G (2013). Association between depression and functional vision loss in persons 20 years of age or older in the United States. JAMA Ophthalmol.

[8] Schönknecht P, Pantel J, Kruse A, Schröder J (2005). Prevalence and natural course of aging-associated cognitive decline in a population-based sample of young-old subjects. Am J Psychiatry.

[9] Lin MY, Gutierrez PR, Stone KL, Yaffe K, Ensrud KE, Fink HA (2004). Study of Osteoporotic Fractures Research Group. Vision impairment and combined vision and hearing impairments predict cognitive and functional decline in older women. J Am Geriat.

[10] Age-Related Eye Disease Study Research Group (2006). Cognitive impairment in the Age-Related Eye Disease Study: AREDS Report No.16. Arch Ophthalmol.

[11] Ong SY, Cheung CY, Li X, Lamoueux EL, Ikram MK, Ding J (2012). Visual impairment, age-related eye diseases, and cognitive function: the Singapore Malay Eye study. Arch Ophthalmol.

[12] Tay T, Wang JJ, Kifley A, Lindley R, Newall P, Mitchell P (2006). Sensory and cognitive association in older persons: findings from an older Australian population. Gerontology.

[13] Lin FR, Metter EJ, O’Brien RJ, Resnick SM, Zonderman AB, Ferrucci L (2011). Hearing loss and incident cognitive impairment. Arch Neurol.

[14] Lin FR, Yaffe K, Xia J, Xue QL, Harris TB, Purchase-Helzner E (2013). Hearing loss and cognitive decline in older adults. JAMA.

[15] Martini A, Castiglione A, Bovo R, Vallesi A, Gabelli C (2014). Aging, cognitive load, dementia and hearing loss. Audiol Neurootol.

[16] Wallhagen MI, Strawbridge WJ, Shema SJ (2008). The relationship between hearing impairment and cognitive function: a 5-year longitudinal study. Res Gerontol Nurs.

[17] Saunders GH, Echt KV (2007). An overview of dual sensory impairment in older adults: perspectives for rehabilitation. Trends Amplif.

[18] Swenor BK, Ramulu PY, Willis JR, Friedman D, Lin FR (2013). The prevalence of concurrent hearing and vision impairment in the United States. JAMA.

[19] Yamada Y, Denkinger MD, Onder G, Henrard JC, van der Roest HG, Finne-Soveri H (2016). Dual sensory impairment and cognitive decline: the results from the Shelter Study. J Gerontol A Biol Sci Med Sci.

[20] National Institute of Population and Social Security Research. Population projections for Japan (January 2012): 2011 to 2060. J Health Welf Stat. Vol. 62, No. 9, 2015/2016.

[21] Tsutui T, Muramatsu N (2005). Care-needs certification in the long-term care insurance system of Japan. J Am Geriatr Soc.

[22] Textbook for eligibility assessment for long-term care insurance 2009, Ministry of Health, Labour and Welfare, 2009 [in Japanese], http://www.mhlw.go.jp/topics/kaigo/nintei/dl/text.2009_3.pdf. Accessed 22 May 2016.

[23] Health, Labour and Welfare Statistics Association. J Health Welf Stat 2014/2015. 2014;62:83–86.

[24] Global Burden of Disease Study 2013 Collaborators (2015). Global, regional, and national incidence, prevalence, and years lived with disability for 301 acute and chronic diseases and injuries in 188 countries, 1990–2013: a systematic analysis for the Global Burden of Disease Study 2013. Lancet.

[25] Gopinath B, Schneider J, McMahon CM, Burlutskv G, Leeder SR, Mitcheil P (2013). Dual sensory impairment in older adults increases the risk of mortality: a population-based study. PLoS One.

[26] Conners MH, Sachdev PS, Kochan NA, Xu J, Draper B, Brodaty H (2015). Cognition and mortality in older people: the Sydney Memory and Ageing Study. Age Ageing.

[27] Sachs G, Carter R, Holtz RL, Smith F, Stump T, Tu W (2011). Cognitive impairment: an independent predictor of excess mortality: a cohort study. Ann Intern Med.

[28] Fisher D, Li CM, Chiu MS, Themann CL, Petersen H, Jonasson F (2014). Age Ageing.

[29] King J, Yourman L, Ahalt C, Eng C, Knight SJ, Pérez-Stable EJ (2012). Quality of life in late-life disability: “I don’t feel bitter because I am in a wheelchair”. J Am Geriatr.

